# Reconsidering the “Classic” Clinical History Associated with Subluxations of the Radial Head

**DOI:** 10.5811/westjem.2019.1.41541

**Published:** 2019-02-14

**Authors:** Kevin Pirruccio, Daniel Weltsch, Keith D. Baldwin

**Affiliations:** * University of Pennsylvania, Perelman School of Medicine, Philadelphia, Pennsylvania; † The Children’s Hospital of Philadelphia, Division of Orthopaedic Surgery, Philadelphia, Pennsylvania; ‡ Tel Aviv University, Sackler Faculty of Medicine, Tel Aviv-Yafo, Israel; § The Chaim Sheba Medical Center at Tel Hashomer, Department of Orthopaedic Surgery, Tel HaShomer, Ramat Gan, Israel

## Abstract

**Introduction:**

The national burden of radial head subluxations in the United States (U.S.) population is poorly defined, and non-classical injury mechanisms have been increasingly reported in recent years. The purpose of this study is to report historical national estimates and demographic characteristics of patients presenting to U.S. emergency departments (ED) with subluxations of the radial head.

**Methods:**

This cross-sectional, retrospective study analyzes the National Electronic Injury Surveillance System (NEISS) database (2001–2017) to identify patients ≤ 7 years of age presenting to U.S. EDs with subluxations of the radial head.

**Results:**

Linear regression (R2 = 0.65; P < 0.01) demonstrated that the annual number of patients presenting to U.S. EDs with subluxations of the radial head increased significantly (P < 0.001) between 2001 (N=13,247; confidence interval [CI], 9,492–17,001) and 2010 (N=21,723; CI, 18,762–24,685), but did not change significantly between 2010 and 2017 (R2 < 0.01; P = 0.85). It also demonstrated that 51.0% (CI, 45.3%–56.6%) of injuries were either self-induced or spontaneous, whereas 36.8% (CI, 31.6%–42.0%) and 9.4% (CI, 8.0%–10.7%) were associated with parents/guardians or siblings, respectively. The majority of injuries occurred in patients who were the age of one (33.5%; CI, 32.1%–35.0%) and two (35.1%; CI, 33.7%–36.6%); females (57.8%; CI, 56.8%–58.9%) were more commonly injured than males.

**Conclusion:**

Although the national burden of radial head subluxations may be less than previously reported, it still results in over 20,000 ED visits annually in the U.S. Given that over half of such injuries are actually self-induced or spontaneous, caretakers should be taught to recognize the clinical presentation of radial head subluxation, since the classically described history of a patient being lifted or pulled by the arm may simply have never occurred.

## INTRODUCTION

The most common pediatric upper-extremity injury is subluxation of the radial head, which may also be termed “pulled elbow” or “nursemaid’s elbow.”^1^,^2^ Mechanistically, this occurs when traction forces act on the hand while the elbow is extended and the forearm is pronated, causing displacement of the annular ligament over the broader head of the radius; often, pain is sufficient to prevent patients from attempting to use their arm.^3^ Injury incidence peaks in children 1–3 years of age, and are rare after age 6 due to thickening of the annular ligament.^2^,^4^ Patients are classically at risk for subluxations of the radial head when being pulled by the arm to be lifted or to be prevented from falling.^5^ In fact, patient histories are often sufficient to make a diagnosis, and treatment – namely, closed reduction of the joint – may be initiated without radiologic imaging as long as there is no clinical suspicion for fracture. However, non-classical injury mechanisms have been widely reported, ranging from falls onto elbows to even spontaneous events during sleep and accidentally having the hands get stuck in fixtures.^6^,^7^

Despite its commonality, the national burden of radial head subluxations in the entire United States (U.S.) population is sparsely defined. Only two nationally representative studies have ever been conducted, to the best of our knowledge.^8^,^9^ Our analysis makes use of more recently available data in order to investigate those studies’ purported radial head subluxation injury trends over the past five years, as well as employing a novel methodology and more strict definition of radial head subluxation to better characterize epidemiological trends and identify new, important demographic characteristics related to the mechanism of injury.

The purpose of this study was, therefore, to report weighted national estimates and demographic characteristics of patients presenting to U.S. emergency departments (ED) between 2001 and 2017 with subluxations of the radial head. Our null, primary hypotheses were that annual national estimates of radial head subluxations have continued to rise in the most recently available data periods, and that injuries would be consistent with classically described mechanisms and histories.

## METHODS

### Data Collection

We performed a retrospective, cross-sectional analysis using the National Electronic Injury Surveillance System (NEISS) database of the U.S. Consumer Product Safety Commission (CPSC) between 2001 and 2017, which describes product- or activity-related injuries presenting to hospital emergency EDs in the U.S. The database is publicly available, de-identified, and published annually; hence, this study was exempt from institutional review board approval. Moreover, it is a nationally representative probability sample of U.S. EDs stratified by size and geographic location, from which reliable, weighted national estimates and sampling errors for queried injuries may be derived.^10^,^11^

### Selection Criteria

In this study, we queried each yearly sample in the NEISS database between 2001 and 2017 for injuries specifically identified as elbow (Body Part Code: 32) dislocations (Diagnosis Code: 55) in patients ≤ 7 years of age. We identified 16,084 such elbow dislocations, translating to 413,198 weighted national estimates of ED visits. Next, we analyzed free-text case narratives for each of these 16,084 visits for explicit diagnoses of radial head subluxations (including “pulled elbow,” “nursemaid’s elbow”). After applying these criteria, 11,647 unique cases remained, amounting to 300,020 weighted national estimates of radial head subluxations presenting to U.S. EDs in patients ≤ 7 years of age during our study period. National estimates, standard errors, and 95% confidence intervals (CI) were derived in Stata/IC 15.1.^12^ We determined significance of trends using adjusted Wald tests. P values < 0.05 (two-sided) were considered significant.

**Population Health Research Capsule**

What do we already know about this issue?

*Classically, clinical histories for patients sustaining radial head subluxations describe a child being pulled by the arm, displacing the annular ligament*.

What was the research question?

Do patients sustaining radial head subluxations demonstrate classically described mechanisms and histories?

What was the major finding of the study?

*Over half of radial head subluxations are self-induced or spontaneous, inconsistent with classical mechanisms*.

How does this improve population health?

*Our findings reduce the negative predictive value associated with non-classical radial head subluxation injury histories for both parents and providers*.

## RESULTS

**Figure 1:**
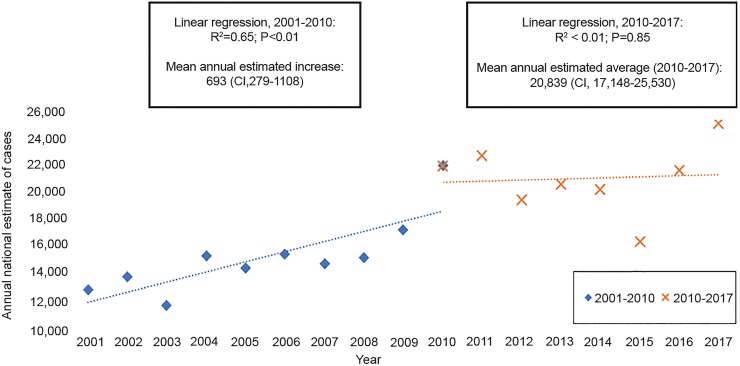
Weighted national estimates of pediatric patients presenting to U.S. emergency departments (EDs) with subluxations of the radial head, 2001–2017. *CI*; confidence interval. This figure overlays two linear regression models for the annual national estimate of radial head subluxations presenting to U.S. EDs between 2001 and 2017. The first linear regression model (blue, dotted line) uses annual national estimates (blue, filled diamonds) from 2001 to 2010 as inputs. The second linear regression model (orange, dotted line) uses annual national estimates (orange cross marks) from 2010 to 2017 as inputs. Results from the linear regression models are shown in the text boxes directly above each model.

The annual number of pediatric patients presenting to U.S. EDs with diagnoses of radial head subluxations increased significantly (P < 0.001) between 2001 (N=13,247; CI, 9,492–17,001) and 2017 (N=24,614; CI, 18,782–30,445) (Table 1). This trend is illustrated graphically in Figure 1, demonstrating that the data best fit a linear regression function (R^2^ = 0.65, p < 0.01) between 2001 and 2010; on average, subluxations of the radial head increased by 693 (CI, 279–1108) cases per year during this period. However, since 2010, there has been no significant change (R^2^ < 0.01; P = 0.85) in the average number of subluxations of the radial head presenting to U.S. EDs annually (20,839; CI, 17,148–24,530).

**Figure 2:**
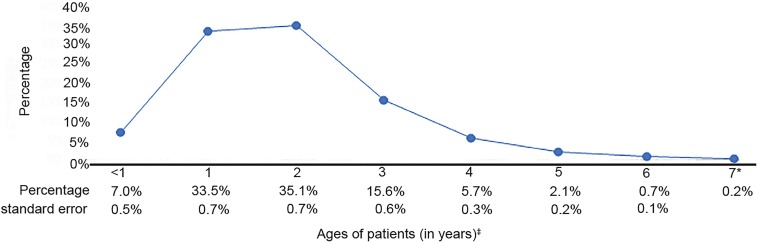
Ages of pediatric patients presenting to U.S. emergency departments with subluxations of the radial head, 2001–2017. *The estimate is considered to be potentially unstable due to the number of unweighted cases from the sample frame totaling <20, the weighted national estimate totaling <1200, or coefficient of variation >33%. Therefore, no standard errors or confidence intervals are provided; the unstable percentage estimate is provided for reference purposes only. Variable results with sample frame totals <20 cases or percentages <0.1% were omitted from this table, resulting in percentage totals not necessarily summing to 100%. ^‡^Age groupings are based on “Age stages defined according to the Eunice Kennedy Shriver National Institute of Child Health and Human Development pediatric terminology” defined by Williams et al. (2012) in paper entitled, “Standard 6: age groups for pediatric trials.”^18^

The ages of patients presenting to U.S. EDs with subluxations of the radial head are shown in Figure 2 with 7.0% (CI, 6.0%–8.0%) of injuries occurring in patients ≤ 1 year of age. About one-third of cases (33.5%; CI, 32.1%–35.0%) occurred in patients who were one year old, and another roughly one-third occurred in those two years old (35.1%; CI, 33.7%–36.6%). Another 15.6% (CI, 14.4%–16.8%) of patients were three years old when sustaining subluxations of the radial head. After age three, the percentage of patients sustaining subluxations of the radial head drops off markedly from 5.7% (CI, 5.0%–6.3%) at age four to 2.1% (CI, 1.7%–2.5%) at age five. Merely 0.7% (CI, 0.4%–1.0%) of injuries occurred in patients six years old, with an insignificant number of injuries occurring at age seven. Table 2 describes the overall demographic characteristics of pediatric patients diagnosed with subluxations of the radial head at a U.S. ED between 2001 and 2017.

The incidence of radial head subluxation was slightly but significantly higher in the summer (27.0%; CI, 26.0%–28.1%) and fall (27.1%; CI, 25.7%–28.5%) than in the winter (21.8%; CI, 20.7%–23.0%) or spring (24.0%; CI, 23.1%–24.9%) (p < 0.001). Importantly, in over half (51.0%; CI, 45.3%–56.6%) of cases, subluxations of the radial head were either self-induced or spontaneous. Additionally, more than one-third of cases (36.8%; CI, 31.6%–42.0%) were associated with parents or guardians handling the patient. Another 9.4% (CI, 8.0%–10.7%) of cases were associated with siblings interacting with the patient, whereas only 4.5% (3.7%–5.4%) of cases were associated with other relatives, caretakers, or friends interacting with the patient. Females (57.8%; CI, 56.8%–58.9%) were more commonly affected than males (42.2%; CI, 41.1%–43.2%). Almost two-thirds of radial head subluxations occurred at home (64.1%; CI, 57.0%–71.2%), while nearly one-quarter occurred in unknown locations (24.0%; CI, 16.7%–31.4%). Effectively all patients were treated in the ED and released from the hospital (99.7%; CI, 99.6%–99.9%).

## DISCUSSION

Our study demonstrates that the national number of radial head subluxations presenting to U.S. EDs has not continued to increase since 2010. Between 2001 and 2010, radial head subluxations presenting to U.S. EDs rose by almost 700 cases per year. In contrast, over the past seven years, this number has steadied at about 21,000 cases per year. Moreover, we found that patients presenting to U.S. EDs with subluxations of the radial head were most often females between the ages of 1–3, with most injuries sustained at home. Lastly, we are the first to report that over half of radial head subluxations were self-induced or occurred spontaneously, rather than associated with parents, guardians, or other individuals handling patients, as is classically reported.

Our annual national estimates of radial head subluxations presenting to U.S. EDs were substantially lower than those previously reported. For example, Brown estimated that between 2005 and 2006, there were about 100,000 annual cases of radial head subluxation presenting to U.S. EDs.^9^ In stark contrast, we found there were only about 15,000 diagnoses of radial head subluxation during these same years. Similarly, Welch, Chounthirath, and Smith found significantly higher estimates of radial head subluxations. In 2001, the authors reported over 20,000 cases presenting to U.S. EDs; in 2011, they reported over 30,000 such injuries.^8^ For comparison, we only observed about 22,000 radial head subluxations presenting to U.S. EDs in 2011. This estimate gap is especially marked when considering that the later study only included patients ≤ 5 years of age, whereas we broadened our population to those ≤ 7 years of age, yet still identified fewer cases.

Given that Welch, Chounthirath, and Smith also queried the NEISS database, we ascribe their substantially higher estimates to two methodological decisions that may have resulted in an overly inclusive definition of injuries constituting radial head subluxations. First, the authors counted *any* patient ≤ 5 years of age sustaining an elbow dislocation as having a subluxation of the radial head.^8^ Therefore, simple dislocations of the humeroulnar joint and mechanisms inconsistent with radial head subluxations are included in their investigation, which has previously been deemed a “common clinical mistake”.^13^–^15^ Second, they included non-dislocation diagnoses in their study if the narrative contained the term “nursemaid’s elbow,” potentially including complex elbow dislocations secondary to fracture – such as Monteggia fractures – inadvertently.^16^ These assumptions may have led to the observed anomaly that, in most years, the authors estimated there were more radial head subluxations than there were total national elbow dislocations in their study population. For instance, in 2011 the authors estimated 30,616 national radial head subluxations in patients ≤ 5 years of age, yet the NEISS database reports only 29,091 total elbow dislocations for this age-matched population.^17^ Therefore, it is likely that this value was inflated.

In contrast, our study excluded all cases for which definitive diagnoses of radial head subluxation were not explicitly made in the narrative sections of cases in which patients were both ≤ 7 years of age, and also coded as having an isolated elbow dislocation. As a result, we consider our findings to represent conservative estimates of radial head subluxation injuries presenting to U.S. EDs, with the advantage of incorporating over five years of recent data to show that the increased trends claimed by the aforementioned studies appear to have leveled off. Regardless, the pediatric health burden of radial head subluxations remains substantial, even under this best-case scenario. The magnitude of these findings implores the use of awareness interventions intended to educate parents, siblings, and other caretakers on safe ways to lift young children and prevent injuries during play. Namely, lifting or swinging young children by the hands and arms should be avoided to prevent primary and recurrent injury; instead, lifting underneath the arms and avoiding forceful “tugs” on the upper extremities can minimize the risk of sustaining a subluxation of the radial head.^6^,^19^

However, our study also suggests there is an equally important, added role for changing the way that parents and guardians are educated about avoiding radial head subluxation injuries in children. Specifically, parents and other caretakers must be taught to recognize the clinical entity of radial head subluxation in order to better determine when medical evaluation and treatment should be pursued, since over half of such injuries occur spontaneously or are self-induced. In other words, considering we found that radial head subluxations with classically described clinical histories – namely, where a parent or other caretaker recounts pulling on the arm of the patient and causing injury – comprise less than half of all cases, it is more likely that an adult may never actually witness the injury occurring; instead, they will have to rely on interpreting symptoms and other conspicuous clinical clues when deciding whether or not an ED visit is merited.^20^ Likewise, these findings may play an important role in changing the way that clinicians in the ED evaluate and manage pediatric upper extremity injuries at the bedside. Our findings reduce the negative predictive value associated with previously non-classical radial head subluxation injury histories, thus maintaining heightened clinical suspicion for radial head subluxation in patients with certain demographic risk factors.

Moreover, our data corroborate previous epidemiological findings that those most at risk for sustaining subluxations of the radial head are often females ≤ 4 years of age, with almost two-thirds of injuries happening while the patient is at home.^4^,^5^,^21^ Similarly, we showed that patients 1–2 years of age constitute the vast majority of those injured. Additionally, the sheer size of our sample allowed us to extend the age limit of our study population in order to calculate that only about 1% of radial head subluxations occurred in those over the age of 5; given the rarity of these injuries in older children, this result is frequently absent from single-institution studies.^22^

## LIMITATIONS

This study has several limitations related to the nature of the NEISS database. First, the accuracy of our analyses depended on the correctness of the narrative sections, which are inherently prone to reporter bias. While such occurrences can never be entirely ruled out, the NEISS employs rigorous data collection methodologies that minimize misdiagnoses and coding errors.^10^,^11^ Second, the NEISS database omits certain clinically relevant variables, including imaging results or the implementation of closed reduction techniques, and does not allow for the determination of primary vs recurrent injury. These variables may have provided information about the successes of various treatment strategies in both the short- and long-term while allowing for nuanced risk-stratification analyses. Most importantly, the dataset only includes injuries that presented to U.S. EDs, and therefore omits cases in which a patient first presented in an outpatient setting, such as a pediatrician’s office or urgent care clinic.^20^,^23^

## CONCLUSION

Subluxation of the radial head is a common early childhood injury of the upper extremity. Prior studies identified an upward trend in the annual number of radial head subluxations presenting to U.S. EDs through 2010, but our analyses show that these estimates may have been inflated and that said trends have largely leveled off in recent years. Even so, we find today that over 20,000 such injuries present to U.S. EDs each year. Furthermore, our study found that the majority of radial head subluxations are self-induced or spontaneous, and often occur at home in children 1–2 years of age. Therefore, it is especially important that caretakers recognize the clinical presentation of radial head subluxation: they may never directly observe the injury occur, and the age of the patient may preclude language skillsets from being developed sufficiently enough to communicate their experience with others. Thus, increased caretaker awareness of these injuries and their presentation may eventually play a substantial role in minimizing this national pediatric health burden.

## Figures and Tables

**Table 1:** Weighted national estimates of pediatric patients presenting to United States emergency departments with subluxations of the radial head, 2001–2017.

Year	National cases	Standard error	95% Confidence interval
2017	24,614	2,934	18,782 – 30,445
2016	21,415	1,759	17,919 – 24,911
2015	16,553	1,399	13,773 – 19,334
2014	20,127	1,646	16,856 – 23,398
2013	20,460	1,689	17,103 – 23,817
2012	19,393	2,083	15,252 – 23,534
2011	22,426	1,855	18,739 – 26,114
2010	21,723	1,490	18,762 – 24,685
2009	17,337	1,382	14,590 – 20,083
2008	15,411	1,165	13,097 – 17,726
2007	15,030	1,274	12,497 – 17,562
2006	15,685	1,573	12,558 – 18,812
2005	14,736	1,413	11,927 – 17,545
2004	15,579	2,098	11,409 – 19,749
2003	12,184	1,671	8,864 – 15,505
2002	14,099	1,786	10,549 – 17,649
2001	13,247	1,889	9,492 – 17,001

**Table 2:** Overall demographic characteristics of pediatric patients presenting to U.S. emergency departments with subluxations of the radial head, 2001–2017.

Demographic variable	Percentage	Standard error	95% Confidence interval
Season			
Summer	27.0%	0.5%	26.0% – 28.1%
Winter	21.8%	0.6%	20.7% – 23.0%
Fall	27.1%	0.7%	25.7% – 28.5%
Spring	24.0%	0.4%	23.1% – 24.9%
Person(s) associated with injury			
Patient (self-induced or spontaneous)	51.0%	2.9%	45.3% – 56.6%
Parent of guardian	36.8%	2.6%	31.6% – 45.0%
Sibling	9.4%	0.7%	8.0% – 10.7%
Other (i.e., relative, teacher, friend)	4.5%	0.4%	3.7% – 5.4%
Unspecified	1.6%	0.2%	1.2% – 2.0%
Sex			
Male	42.2%	0.5%	41.1% – 43.2%
Female	57.8%	0.5%	56.8% – 58.9%
Race			
White	48.5%	3.9%	40.8% – 56.2%
Black	10.3%	1.9%	6.5% – 14.1%
Other	3.0%	0.8%	1.4% – 4.6%
Asian^a^	1.7%		
Hispanic	9.2%	1.9%	5.4% – 13.0%
Race not specified	26.9%	4.2%	18.5% – 35.4%
Treated and released from hospital	99.7%	0.1%	99.6% – 99.9%
Location			
Unknown	24.0%	3.7%	16.7% – 31.4%
Home	64.1%	3.6%	57.0% – 71.2%
Street^a^	0.2%		
Public	4.0%	0.3%	3.4% – 4.5%
School	3.0%	0.2%	2.6% – 3.4%
Sports	4.7%	0.5%	3.7% – 5.6%

a The estimate is considered to be potentially unstable due to the number of unweighted cases from the sample frame totaling < 20, the weighted national estimate totaling < 1200, or coefficient of variation > 33%.

Therefore, no standard errors or confidence intervals are provided; the unstable percentage estimate is provided for reference purposes only. Variable results with sample frame totals < 20 cases or percentages < 0.1% were omitted from this table, resulting in percentage totals not necessarily summing to 100%.
